# Combined RP-HILIC for suspect screening of persistent, mobile, and toxic substances in surface water: A case study

**DOI:** 10.1038/s41598-025-29664-1

**Published:** 2025-12-05

**Authors:** Javad Mottaghipisheh, Rajneesh Gautam, Lutz Ahrens

**Affiliations:** Department of Aquatic Sciences and Assessment, Swedish University of Agricultural Sciences (SLU), PO Box 7050, SE-75007 Uppsala, Sweden

**Keywords:** High-resolution mass spectrometry, Hydrophilic interaction liquid chromatography (HILIC), Reversed-phase (RP), Suspect screening, Surface water, Wastewater water treatment plant (WWTP), Chemistry, Environmental sciences, Water resources

## Abstract

Persistent, Mobile, and Toxic (PMT) and very Persistent and very Mobile (vPvM) substances pose a significant environmental threat due to their widespread distribution, persistence, and potential toxicity. While previous studies have used reversed-phase (RP) and hydrophilic interaction liquid chromatography (HILIC) to extend analyte coverage, this study uniquely integrates a dual-chromatographic approach with multi-sorbent solid phase etration (SPE) and machine-learning-based retention time indices to rigorously minimize false positives and enhance confidence in suspect screening of PMT/vPvM substances. Two independently optimized LC methods (RP and HILIC) were applied in parallel to the same SPE extract to identify potential chromatographic blind spots. We applied this robust framework to systematically screen surface water and wastewater treatment plant (WWTP) effluent across a water catchment in Uppsala, Sweden. The RP and HILIC workflows, applied as independent chromatographic runs, identified 84 compounds after multi-stage filtering (with retention time indices (RTI) correction applied only to the RP data), with 27% and 48% uniquely detected by HILIC and RP, respectively, highlighting analytical blind spots overcome by this integrated approach. Strategic site selection, encompassing upstream rural zones, on-site sewage facilities, and major WWTP discharge points, demonstrated how local land use impacts PMT/vPvM profiles. This comprehensive method provides a powerful tool for environmental monitoring and regulatory surveillance of PMT/vPvM compounds.

**Supplementary Information:**

The online version contains supplementary material available at 10.1038/s41598-025-29664-1.

## Introduction

Persistent, Mobile, and Toxic (PMT) and very Persistent, very Mobile (vPvM) substances have emerged as significant environmental contaminants due to their ability to persist and travel long distances in aquatic systems^1,2^. These substances, characterized by their resistance to degradation and polar nature, present substantial challenges for environmental monitoring and risk assessment. Their high mobility in water systems and limited removal during wastewater treatment make them a concern, as they can accumulate in surface and groundwater, posing risks to ecosystems and human health^3^.

Unlike Persistent, Bioaccumulative, and Toxic (PBT) substances, PMT/vPvM compounds do not accumulate in sediments or biota, making their environmental behavior and risks distinct^4^. Their mobility and persistence in water bodies necessitate dedicated identification strategies to prevent long-term contamination of drinking water sources.

Wastewater treatment plants (WWTPs) and on-site sewage facilities (OSSFs) have been reported to influence the distribution and behaviour of contaminants such as pharmaceuticals, personal care products, pesticides, and per- and polyfluoroalkyl substances (PFASs) in aquatic environments^5^. While WWTPs typically maintain stable contaminant profiles, OSSFs display greater variability in micropollutant release, highlighting their diverse effects on water quality^5^. Recognizing the environmental significance of PMT/vPvM substances, the European Commission has emphasized the need for systematic investigations to develop robust methodologies for their detection and management^6^.

Advancements in analytical chemistry, particularly suspect screening methodologies combined with high-resolution mass spectrometry (HRMS), have revolutionized the detection of emerging contaminants^7^. Unlike targeted analyses, which require prior knowledge of analytes, suspect screening enables the identification of unknown or poorly characterized compounds. This approach uses suspect lists derived from regulatory databases, market data, and environmental studies to screen for potential contaminants^8^.

Detecting and identifying PMT/vPvM substances remain challenging due to their polar and hydrophilic characteristics, which often fall outside the scope of conventional analytical techniques^6^. Reverse-phase liquid chromatography (RP-LC), commonly used in environmental analysis, is optimized for non-polar or moderately polar compounds but performs poorly with highly polar analytes^2^. Reemtsma and Jekel (2006) provide a foundational overview of polar organic pollutants in water, highlighting analytical challenges and the need for polarity-extended methods in environmental screening^9^. This limitation leaves many PMT/vPvM substances undetected, creating an analytical gap^10^. Hydrophilic interaction liquid chromatography (HILIC) offers a promising alternative for such analytes, providing improved retention and separation of polar compounds by leveraging interactions with their stationary phases^11^. When combined, HILIC and RP-LC can serve as complementary techniques, providing a broader analytical scope for studying complex environmental matrices^12,13^. However, most previous studies have applied these techniques separately or in complementary workflows, which limits the ability to systematically quantify the analytical blind spots of each method.

Serial RP–HILIC chromatography, often referred to as polarity-extended chromatography, has been extensively developed and utilized to broaden polarity coverage in single-run environmental screening and omics analyses^14,15^. These studies have demonstrated the method’s capability to comprehensively detect analytes ranging from highly polar to non-polar compounds. For example, Haggarty et al. (2015) demonstrated that serially coupled RP-HILIC with simultaneous gradients enhances metabolomic coverage by enabling the separation of non-polar, polar, and highly polar compounds in one workflow^16^.

Although RP-HILIC methods offer greater flexibility through independent optimization of retention mechanisms and often provide superior resolution for structurally diverse compounds, Montes et al. (2022) recommended combining mixed-mode LC (MMLC) and RP chromatographic modes to maximize analyte coverage—particularly when supercritical fluid chromatography (SFC) is unavailable and HILIC is not included. Their suspect screening approach, applied to surface and wastewater samples across Northern Portugal and Galicia, led to the tentative identification of 343 contaminants of emerging concern (CECs), including 153 classified as persistent, mobile, and toxic (PMT) and 23 as very persistent and very mobile (vPvM), highlighting the effectiveness of this dual-mode strategy for broad environmental monitoring^17^. However, previous studies did not apply RP and HILIC in parallel, limiting their ability to directly assess the selectivity gaps or retention limitations of each method.

In this study, two independently optimized LC methods (RP and HILIC) were applied in parallel to the same solid phase extraction (SPE) extract to quantify chromatographic blind spots. By combining this dual-mode approach with multi-sorbent SPE, high-resolution mass spectrometry (HRMS), and machine-learning-based retention time indices (RTI), we enhance compound confirmation and minimize false positives. This comprehensive strategy addresses critical analytical gaps in PMT/vPvM monitoring and supports regulatory efforts to improve surveillance of these substances. Additionally, our sampling design—covering upstream rural zones, small-scale OSSFs, and large-scale WWTP discharge points—provides insights into how land use influences PMT/vPvM profiles, offering transferable knowledge for other mixed-use catchments across Europe and globally. This workflow enables environmental laboratories to benchmark and reduce RP–HILIC blind spots in suspect screening.

## Materials and methods

### Sampling and sample Preparation

**Fig. 1:**
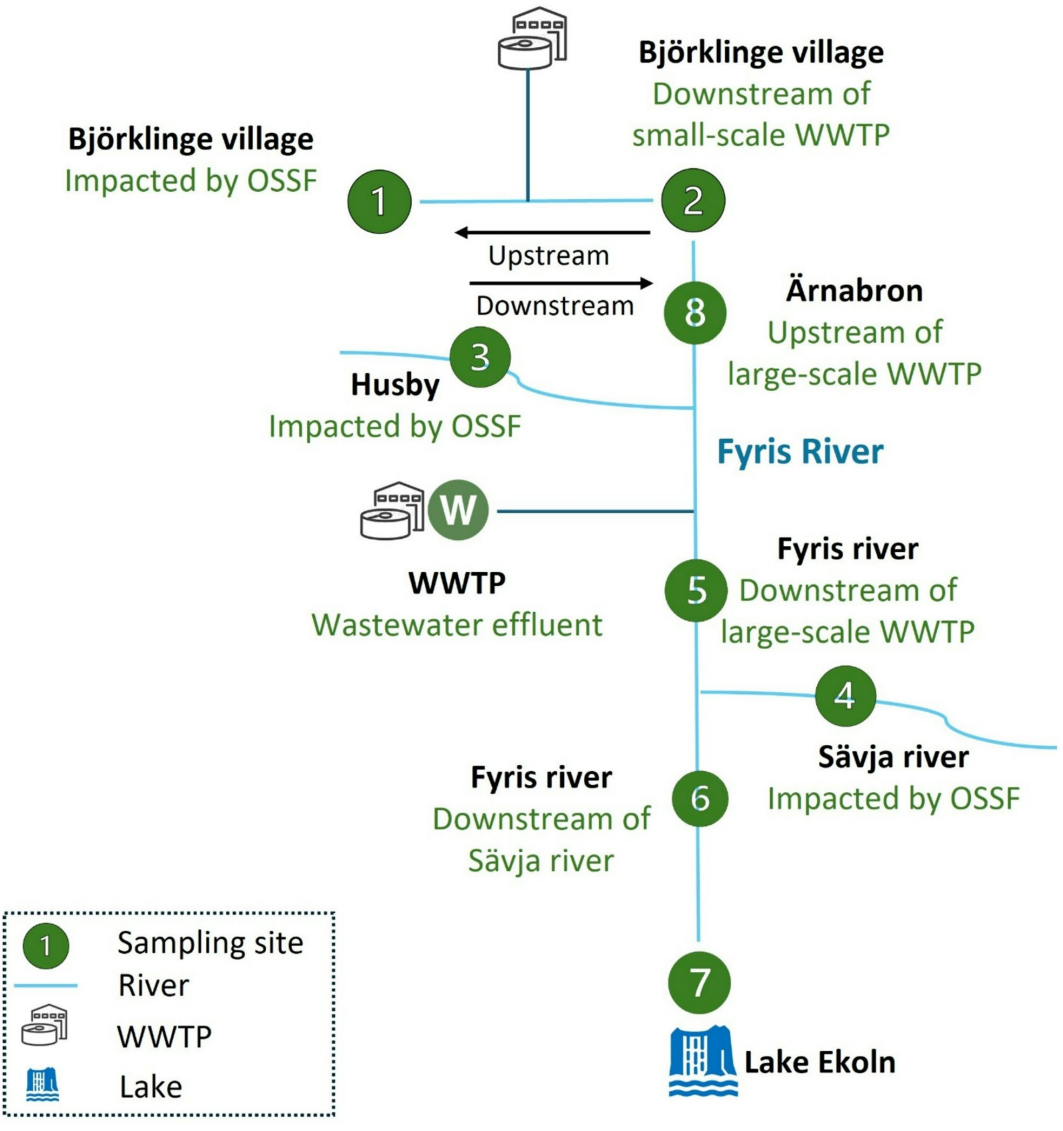
Sampling locations across surface water and wastewater systems impacted by wastewater treatment plants (WWTP) and on-site sewage facilities (OSSFs) in Uppsala, Sweden.

Surface water samples (*n* = 8) were collected from diverse locations along the Fyris River catchment and Lake Ekoln, as well as WWTP effluent at Uppsala, Sweden, including surface water and wastewater systems, representing urban, industrial, and rural influences (Fig. 1, Figure S1, Table S1 in Supporting Information (SI1)). These sites were selected to capture a range of contamination profiles influenced by urban, industrial, agricultural, and semi-natural land uses, providing a representative cross-section of typical European catchments impacted by full-scale WWTPs, small-scale OSSFs and diffuse sources.

Samples (1 L) were collected in triplicate in pre-cleaned high-density polyethylene (HDPE) bottles, filtered using 0.7 μm glass fiber filters (Whatman GF/F) to remove particulate matter, and stored at 4 °C in pre-cleaned polypropylene containers to minimize adsorption and degradation of polar and hydrophilic PMT/vPvM substances^18^. Procedural blanks and field blanks were prepared to monitor potential contamination during sample handling and processing. Each sample, procedural blank, and pooled quality control (QC) was spiked prior to extraction with 50 µL of a mixed isotopically-labelled PFAS internal-standard solution (0.05 µg/mL, Table S2 in SI1). In this study the IS served as process and QC markers for ESI(−) (RP and HILIC) and used to anchor retention times (RT), monitor within-/cross-batch drift, and check carryover.

Solid-phase extraction (SPE) was carried out for analyte enrichment following a previously described protocol^8^. In brief, SPE was performed on 500 mL of each sample in triplicate using four different SPE materials: Oasis HLB, Strata-X-AW, and Strata-X-CV (Phenomenex, Torrance, USA) and Isolute ENV+ (Biotage, Ystrad Mynach, UK). These sorbents were packed into 6 mL polypropylene SPE tubes fitted with 20 μm frits to create an in-house cartridge configuration, allowing simultaneous extraction of a broad range of polar and non-polar compounds. Samples were then eluted with 4 mL of methanol/ethyl acetate (50:50, v/v) containing 2% ammonia, selected to promote desorption of both acidic and basic compounds from the sorbents and enhance recovery across a broad polarity range. The eluates were evaporated under a gentle nitrogen stream and reconstituted in methanol/ethyl acetate (50:50, v/v) containing 1.7% formic acid to stabilize analytes and improve ionization efficiency during LC-HRMS analysis. Importantly, the same extraction protocol was applied for both RP and HILIC to ensure comparability between the two separation techniques. Using different extraction methods would compromise the ability to directly compare their performance. The reconstituted extracts were filtered through 0.45 μm syringe filters and stored at − 20 °C prior to instrumental analysis.

### Instrumental analysis

Liquid chromatography analysis was performed using a Vanquish Horizon ultra-performance liquid chromatography (UPLC) system (Thermo Fisher Scientific, Bremen, Germany). Two complementary chromatographic methods (i.e. RP and HILIC) were employed to provide a comprehensive analytical scope.

For RP-LC, a Waters CORTECS C18 + column (90Å, 2.7 μm, 2.1 mm × 100 mm) was used to target non-polar and moderately polar compounds. The mobile phases consisted of water with 0.1% formic acid as solvent A and methanol with 0.1% formic acid as solvent B in positive ionization mode. On the other hand, water with 5 mM ammonium acetate (solvent A) and methanol with 5 mM ammonium acetate (solvent B) were used in negative ionization mode. A gradient elution method was applied, starting at 10% solvent B, ramping to 90% over 16 min, holding for 2 min, and returning to 10% for re-equilibration within a 20 min runtime. The flow rate was maintained at 0.3 mL/min, with an injection volume of 10 µL.

For HILIC, a Waters CORTECS HILIC column (2.7 μm, 3 mm × 100 mm) was employed to target polar compounds. This high-efficiency, solid-core column operates *via* hydrophilic interactions, primarily partitioning and hydrogen bonding, offering orthogonal selectivity compared to RP and enhanced retention and sensitivity for highly polar analytes. The mobile phases consisted of water: acetonitrile (95:5) with 5 mM ammonium formate as solvent A, and acetonitrile-water (95:5) with 5 mM ammonium formate as solvent B were used for analyzing samples in both positive and negative ionization modes. A gradient method started at 100% solvent B, decreased to 5% over 20 min, held for 5 min, and returned to 100% over a total runtime of 30 min. The flow rate was set to 0.7 mL/min with an injection volume of 10 µL.

Mass spectrometric analysis was conducted using the QExactive Focus Orbitrap mass spectrometer equipped with a heated electrospray ionization (HESI-II) source. The system operated in both positive and negative ionization modes to maximize the coverage of analytes. Full MS scans were performed across a mass range of 80–1000 m/z for RP-LC and 100–1000 m/z for HILIC at a resolution of 70,000 (at m/z 200). Data-dependent acquisition (DDA) mode was used to collect MS/MS spectra for the most intense ions, employing stepped collision energies of 10, 20, and 40 eV. The MS/MS spectra were acquired at a resolution of 17,500. The ion source settings were optimized for sensitivity and included a sheath gas flow rate of 35 a.u., auxiliary gas flow rate of 10 a.u., spray voltage of 3.0 kV, capillary temperature of 350 °C, and auxiliary gas heater temperature of 300 °C.

### Data processing and identification workflow

#### Suspect list, compound discoverer workflow, and data analysis

This study focused on a list of 318 prioritized PMT/vPvM substances from the NORMAN Network dataset (NORMAN-SLE-S36.0.3.0, updated 2022) registered under REACH^19^. These substances were classified into five main categories: 206 industrial chemicals, 42 pharmaceuticals, 39 pesticides, 23 PFAS, and 8 personal care products. The distribution of compound classes reflects the composition of this curated list rather than general environmental prevalence. All information regarding the compounds’ SMILES, molecular formula, molecular weights, categories, applications, and their physicochemical and toxicological data are shown in Table S1 in SI2. Toxicological data, including acute toxicity thresholds for fish, daphnids, mysids, green algae, and log *n*-octanol-water partition coefficient (K_ow_) and water solubility properties, were calculated using the Ecological Structure-Activity Relationships (ECOSAR) program 2.2.^20^.

Data processing was performed using Compound Discoverer 3.3 (Thermo Fisher Scientific), with the specific steps of the workflow summarized in Table S3 in SI1. In brief, this workflow facilitated the detection, annotation, and comparison of unknown compounds across samples. RT alignment was performed separately for each chromatographic mode (RP and HILIC) using a conservative ± 2.0 min cross-batch matching window with ± 5 ppm mass tolerance. These parameters were chosen after following QC evaluation: internal standards (IS), RTI calibrants, and system-suitability test (SST) injections showed tight within-batch RT reproducibility but small, systematic cross-batch/matrix drifts. Thus a narrower RT filter risked excluding true features across batches. RT was therefore used only as a prefilter and final feature annotations additionally required accurate mass, consistent isotopic patterns, and MS/MS spectral agreement. Low signal(S)/noice (N) or aberrant isotope-pattern features were removed. We also applied an RTI workflow to normalize sample-to-sample RT differences and to imporve candidate ranking. In ESI(−), PFAS IS acted as RT anchors and drift checks prior to spectral matching; in ESI(+), alignment relied on pooled-QC/SST injections, blanks, and RTI calibrants. Accordingly, positive-mode signals were reported as qualitative or relative unless confirmed by MS/MS data.

Suspect screening was performed to identify PMT compounds from the prioritized list, using multiple databases including mzCloud, mzVault, ChemSpider, ChEMBL, ECHA, EPA DSSTox, EPA ToxCast, MassBank, and PubMed. Compound annotation was supported by the mzLogic algorithm, which ranks candidates based on structural similarity. Background signals were subtracted, and gap filling was used. We did not apply log D filters for HILIC separation; instead log D (pH 7) was used only as a plausibility check, and confidence was based on ± 5 ppm mass error tolerance, mode-specific RT windows (RP 0–20 min; HILIC 0–35 min), isotopic pattern matching, MS² library/in-silico matching, and RTI for RP, and reported using Schymanski levels.

Integration of RP-LC and HILIC data was central to this approach, allowing cross-validation of compounds identified by both methods to enhance detection confidence. Additionally, the unique detections from each method highlighted their complementary roles, addressing analytical blind spots and ensuring comprehensive coverage of PMT/vPvM substances.

#### Retention time indices (RTI)

The RTI for LC-HRMS (version 2.5.0), developed by the National and Kapodistrian University of Athens, Greece, was utilized to enhance the reliability and accuracy of compound identification by minimizing residual errors between experimental and predicted retention time (tR) *via* removing false positive features^21^. The advanced machine learning algorithm OTrAMS (Ordered Traits and Machine Learning System) model was employed to address this^22^. Calibration was achieved using RTI calibrants, encompassing four mixtures of 18 compounds each, tested under + ESI and -ESI modes (for details, see Figures S2 and S3, Tables S4 and S5 in SI1). The calibration method employs the RTI formula as shown in the Eqs. 1 and 2.

1 $$RTI = \:{\frac{{tR}_{x}-{tR}_{min}}{{tR}_{max-}{tR}_{min}}}_{}x\:1000\:$$

2 $$RTI = {\alpha} \:\left({tR}_{c}\right)+C$$

where, *α* is defined as calibration coefficient, $\:{tR}_{x}$ and $\:\:{tR}_{c}$ as RT observed for the calibrants and a compound, respectively, and $\:{tR}_{min}$ and $\:{tR}_{max}$ are the minimum and maximum tR observed for the calibrants, respectively. This standardized approach confirms stability across analytical runs in the RP separation technique, enhances confidence in tR predictions, and supports more robust compound annotation in our study. Based on the RTI workflow, compounds are categorized into four confidence levels. The highest tR matches are classified from Level 1 to Level 4 (box 1–4, described at Table S6 in SI1), while Level 4 is considered as false-positive. Compounds at Level 2 are also acceptable, though with lower confidence than Level 1 (Table S6 in SI1).

For compound identification, the in silico fragmentation tool in Compound Discoverer 3.3, FISh (Fragment Ion Search), was utilized, along with matching the scores within different mass spectral libraries. To further enhance confidence, all MS² spectra were verified using CFM-ID 4.0 (Competitive Fragment Modeling Identification)^23^ and the open mass library MassBank (https://www.massbank.eu, Last Updated version on 2024.11)^24^, ensuring precise structural annotation and improved reliability in compound identification. For the compound confidence level assessment, we adapted criteria from the Schymanski classification^25^. Accordingly, Level 1 compounds were confirmed with reference standards (RT and MS²). When MS² data were unavailable, features were assigned to Level 5 (accurate mass only), Level 4 (accurate mass and an unequivocal molecular formula), or Level 3.3 (accurate mass and molecular formula plus a match to a plausible candidate in mass/suspect libraries). When MS² data were available, stricter criteria were applied: Level 3.2 (low-quality MS²; low matching scores in Compound Discoverer; FISh < 50, less diagnostic fragments), Level 3.1 (higher CD matching scores, FISh ≥ 50, more diagnostic fragments, but not fully concordant with MassBank and the in-silico fragmentation tool CFM-ID), Level 2.2 (high-similarity library MS² scores in CD with full agreement from MassBank and CFM-ID, but isomeric uncertainty remains), and Level 2.1 (high-similarity library MS² with MassBank/CFM-ID support and no isomeric alternatives).

### Statistical analysis

Spatial similarity among sites were quantified by computing pairwise Spearman correlations (ρ) on log 10-transformed intensities (RP and HILIC data collapsed per compound as the site-wise maximum). The resulting correlation matrix was visualized with hierarchical clustering (average linkage). For community-level composition, non-metric multidimensional scaling (NMDS) was applied using Bray–Curtis on Hellinger-transformed data. The statistical analysis were performed using RStudio (V 2024.12.0 + 467), with the corresponding R script provided (Table S2 in SI2).

## Results and discussion

### Identification of compounds using RP and HILIC

**Fig. 2:**
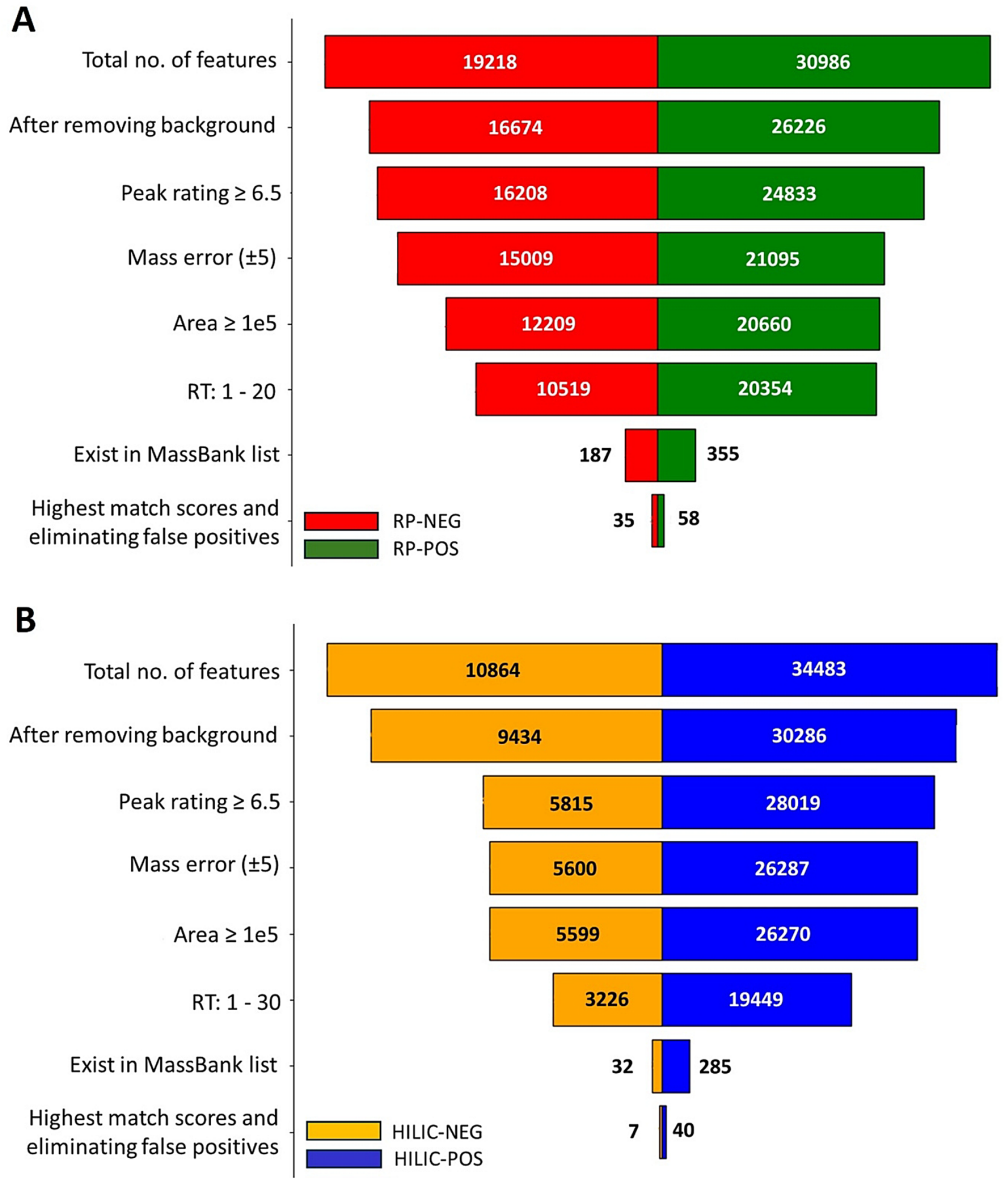
Number of features treated using (**A**) RP and (**B**) HILIC application. For details see Tables S8-S10 in SI.

The dataset reveals the distribution and refinement of chromatographic features in RP and HILIC techniques in both + ESI (POS) and -ESI (NEG) ionization modes (Tables S7 in SI1 and S3─S5 in SI2). Figure 2 and Table S7 (SI1) demonstrate the stepwise reduction of features through quality assurance (QA)/QC filtering and the subsequent pre-annotation prioritization based on suspect and library matching. Applying filtering, including background exclusion, peak rating, mass tolerance, and RT detection windows, resulted in a significant reduction of features to 355 (RP-POS), 187 (RP-NEG), 285 (HILIC-POS), and 32 (HILIC-NEG) (Fig. 2). Subsequent application of criteria selecting features with the highest match scores (mzCloud, mzVault, and FISh) and eliminating false positives (Fig. 2, Table S7 in SI1) reduced the features finally to 58 (RP-POS), 35 (RP-NEG), 40 (HILIC-POS), and 7 (HILIC-NEG) (Fig. 2). All features detected as having MS2 were checked through MassBank and CFM-ID 4.0^23^. Finally, incorporating the RTI filter improved specificity beyond fixed RT windows. In RP mode, RTI removed 33 of 93 pre-RTI candidates (− 35%), predominantly at tentative levels (Level 3.2 reduced 41→28; Level 3.3 reduced 32→17), while preserving all compounds at Level 1 and 2 (Tables S3 and S4 in SI2). Of the 60 RP assignments retained after RTI, 35 fell within the strict RTI acceptance domain (box 1, described at Table S6 in SI1) and 25 were retained as box 2 (described at Table S6 in SI1) due to consistent MS/MS and mass accuracy despite modest RT deviations (Table S4 in SI2).

The separate applications of HILIC and RP provided complementary insights into compound identification by leveraging the distinct selectivity of each chromatographic mode. In total, 84 unique compounds were determined after combining both ionization modes within each chromatographic technique. HILIC identified 23 unique polar compounds (Table S6 in SI2), typically challenging for RP to retain, demonstrating its strength in analyzing hydrophilic analytes with functional groups such as hydroxyl and amine. Examples include dimorpholinodiethyl ether, DMH (5,5-dimethylhydantoin), hexamethyldisiloxane, losartan, methylpiperazine, m-phenylenediamine, pymetrozine, skatole, sulfadimidine, and triflumezopyrim. In contrast, RP identified 40 unique compounds (Table S4 in SI2), such as oxcarbazepine, perfluorohexanoic acid, and galaxolide, which are generally more hydrophobic and exhibit higher logP values. Both methods confirmed 21 overlapping compounds (Table S5 in SI2), including erythromycin, 1,2,3-benzotriazole, 2,6-xylidine, acid red 337, azobisisobutylonitrile, candesartan, ibuprofen, imazapyr, and isophorone diisocyanate. These overlapping compounds typically possess intermediate polarity or amphiphilic properties, allowing retention in both chromatographic systems. These findings reinforce the complementary nature of RP and HILIC and support their parallel use to achieve broader chemical coverage and reduce analytical blind spots, further enhanced by spectral tools and RTI for greater confidence. To quantify polarity differences, log P values were compiled for the identified compounds (dimensionless). Log P values were available for **77** compounds (8 entries were salts or dyes and lacked values), and were summarized by detection category. HILIC-only identifications (*n* = 22 with log P) had a median log P of 1.7 (IQR: −0.15–3.00), whereas RP-only identifications (*n* = 35) had a median log P of 2.6 (IQR: 1.05–3.50). Compounds detected by both techniques (*n* = 20) showed intermediate polarity with a median log P of 1.4 (IQR: 0.70–2.15). On average, the RP-only set was ~ 0.9 log P units more hydrophobic than the HILIC-only set, supporting the expected and complementary selectivity of the two chromatographic modes. For transparency, a “log P” column has been added to Table S6 in SI2.

Unlike previous studies that applied RP or HILIC separately, we used both in parallel as independent runs to directly quantify each method’s analytical blind spots. This highlights the critical need for complementary workflows to avoid underestimating PMT/vPvM substances. For example, Schulze et al. (2019) combined multiple chromatographic (MMLC, HILIC, RP, and SFC) with various SPE techniques to detect 43 of 64 targeted persistent and mobile organic chemicals (PMOCs), but their methods were complementary rather than parallel, limiting direct assessment of method-specific limitations^26^. Similarly, Castro et al. (2021) showed that combining different sampling (POCIS, SPE) and chromatographic modes (RP, MMLC) significantly improves detection coverage, identifying 343 compounds, including 153 PMT and 23 vPvM substances^4^. However, their workflow also lacked a systematic quantification of blind spots for each chromatographic mode. In contrast, our parallel RP-HILIC strategy enables a more rigorous evaluation of selectivity, offering clearer insights for improving analytical coverage and reliability in PMT/vPvM monitoring. Because our suspect list is intentionally enriched for industrial chemicals (206 of 318 entries; Table S1 in SI2), raw across-class frequencies should not be interpreted as indicators of environmental prevalence. Accordingly, we focus on site-to-site contrasts and the complementary coverage provided by parallel RP–HILIC rather than absolute cross-class comparisons.

### Identified compounds using RP

**Level 1**

Level 1–2 identifications are highlighted in the main text, whereas Levels 3–5 are provided in the SI1 with corresponding evidence. Of the 93 identified compounds using the RP technique (Tables S3 and S4 in SI2), metformin **(**C_4_H_11_N_5_, m/z 130.1086 [M + H]^+1^) was identified at Level 1 by confirming with the reference standard and MS² match. This antidiabetic exhibited persistence in aquatic environments, highlighting its prevalence in WWTP and the aquatic environment^27^. Its short tR (0.78 min) using RP agreed with the RTI prediction, demonstrating effective capture of highly polar analytes in RP (Figure S4a in SI1).

**Level 2**

At Level 2, 5 compounds were identified. Two Level 2.1 compounds—isophorone diisocyanate (IPDI) and piperazine—were supported by concordant RTI and MS² (FISh/CFM-ID; Figure S4b–c in SI1). IPDI, (C_12_H_18_N_2_O_2_, m/z 223.1439 [M + H]^+1^**)**, used in polyurethane production^28^ and classified as a respiratory sensitizer by the U.S. Environmental Protection Agency (EPA)^29^, was detected in both RP and HILIC modes, with higher intensity in RP and maximal intensity in wastewater effluent (Figure S4b in SI1), indicating incomplete removal during treatment. Piperazine (C_4_H_10_N_2_, m/z 87.09155 [M + H]^+1^), an industrial and pharmaceutical intermediate with known antinematodal activity^30^ showed diagnostic fragments (m/z 70.065, 56.049) with RTI agreement. Three Level 2.2 compounds, N,N′-diphenylguanidine (a rubber accelerator previously detected in industrial wastewater^31^, N-2-ethylhexyl bicycloheptenedicarboximide (an insect repellent^32^, and N-butyl benzenesulfonamide (a plasticizer frequently reported in aquatic environments^33^, had high MS² matches with acceptable RTI (box 2, described at Table S6 in SI1) (Figure S4d–f in SI1).

**Level 3**

At Level 3.1 (*n* = 2), allyl 2,3-epoxypropyl ether (C_6_H_10_O_2_, m/z 115.0752 [M + H]^+^) and azithromycin (C_38_H_72_N_2_O_12_, m/z 375.2612 [M + H]^+^) exceeded FISh thresholds and showed strong agreement with both spectral library and in-silico matches, but structural ambiguity prevented higher confidence assignment. Allyl 2,3-epoxypropyl ether is a toxic and potentially carcinogenic epoxy ether^34^, while azithromycin—frequently detected in hospital effluents—indicates environmental persistence and potential ecotoxicity^35,36^. Level 3.2 (*n* = 28) comprised tentatives with lower FISh/library scores, yet acceptable experimental/predicted tR alignment (RTI Level 1) or within the applicability domain (RTI Level 2). Representative pharmaceuticals include candesartan (C_24_H_20_N_6_O_3_), climbazole (C_15_H_17_ClN_2_O_2_), diclofenac sodium (C_14_H_11_Cl_2_NO_2_), ibuprofen (C_13_H_18_O_2_), phenazone (C_11_H_12_N_2O_), phenytoin (C_15_H_12_N_2_O_2_), and iohexol (C_19_H_26_I_3_N_3_O_9_). Level 3.3 (*n* = 17) contained lower-confidence tentatives such as erythromycin (C_37_H_67_NO_13_), atrazine (C_8_H_14_ClN_5_), and diuron (C_9_H_10_Cl_2_N_2_O), all widely reported in aquatic environments^37^, and nonetheless captured by the combined RP–HILIC workflow, underscoring its value for broad contaminant screening.

**Levels 4 and 5**

Two compounds, including 4-(1-phenylmethyl)-1,3-benzenediol (C_14_H_14_O_2_) and 1-ethyl-2,3,3-trimethyl-3 H-indolium iodide (C_13_H_19_N), were identified at Level 4, as both cases matched the molecular weight and exact mass, and were validated using the RTI method. Lastly, Level 5 included five compounds characterized by their precise mass.

### **Identified compounds using the HILIC separation technique**

**Level 1**

Among 47 HILIC identifications (Table S5 in SI2), metformin was confirmed at Level 1 using a reference standard (Figure S5a in SI1). Notably, HILIC gave ~ 16× higher metformin intensity than RP, underscoring HILIC’s suitability for identification/quantification of this compound across matrices^38^.

**Level 2**

Three compounds, 5,5-dimethylhydantoin (DMH, C_5_H_8_N_2_O_2_, m/z 129.06581 [M + H]^+1^), (C_12_H_24_N_2_O_3_, m/z 245.18612 [M + H]^+1^), and 4-aminophenol (C_6_H_7_NO, m/z 110.0601 [M + H]^+1^), were assigned Level 2.1 based on top-ranked MS^2^ matches (libraries, FISh, CFM-ID) (Figure S5b–d in SI1). DMH, used as a preservative/disinfectant and pharmaceutical intermediate, has generally low reported toxicity but warrants qualitative dietary risk assessment (inclusing its degradate EMH) and can originate from industrial/biocidal uses or DBDMH hydrolysis^39–41^. Dimorpholinodiethyl ether, an industrial blowing agent associated with dermatotoxicity^42^, showed fragments m/z 158.11757 (C_8_H_16_NO_2_^+^), m/z 114.09135 (C_6_H_12_NO^+^), and m/z 96.08080 (C_6_H_10_N^+^)^19^ (Figure S5c in SI1). 4-Aminophenol, a toxic paracetamol by-product with reported aquatic releases, exhibited a characteristic MS2 spectrum showing fragmentations corresponding to the loss of hydroxyl group (C_6_H_6_N^+^, m/z 92.04947), and the opened anilin moiety (C_5_H_8_N^+^, m/z 82.06516), and 67.04165 (C_4_H_5_N^+^) (Figure S5d in SI1).

**Level 3**

Among 36 tentative HILIC identified compounds, skatole (C_9_H_9_N, RT: 8.15 min, m/z 132.08086 [M + H]^+1^) was the only Level 3.1 compound: Its MS^2^ spectrum matched in-silico predictions and libraries, but structural ambiguity with a near isomer prevented higher confidence. Skatole is widely used as a flavor/fragrance and is frequently reported in surface waters^43^. Most Level 3.2 compounds were pharmaceuticals, including ibuprofen (C_13_H_18_O2, m/z 205.12369 [M-H]^[- [1^), iohexol (C_19_H_26_I_3_N_3_O_9_, m/z 821.8881 [M + H]^+1^), losartan (C_22_H_23_ClN_6_O, m/z 423.16956 [M + H]^+1^), phenazone (C_11_H_12_N_2_O, m/z 189.10229 [M + H]^+1^), phenytoin (C_15_H_12_N_2_O, m/z 253.0974 [M + H]^+1^), all previously observed in aquatic environments^44^. An additional 14 suspects were assigned at Level 3.3 based on accurate mass/formula and library matches but lacked confirmatory MS^2^ evidence.

**Levels 4 and 5**

Two PMT candidates were assigned at Level 4 (isobaric formulas with exact-mass agreement), and three additional contaminants were Level 5 (exact-mass only). These results support integrating dual-mode LC–HRMS (RP + HILIC) into regulatory monitoring to improve detection of PMT/vPvM substances that are often overlooked by conventional single-mode workflows.

### Spatial distribution

**Fig. 3:**
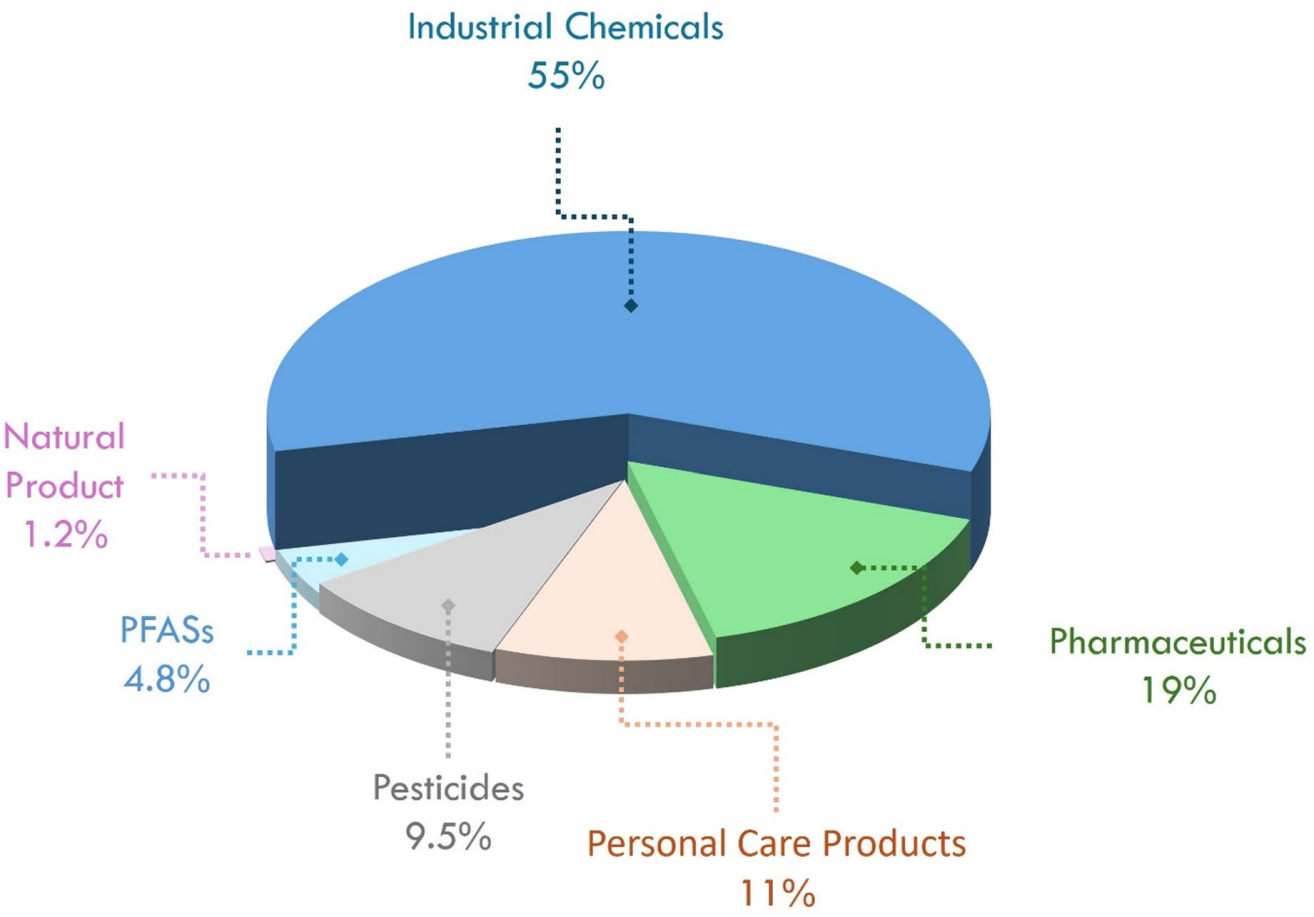
Categories of the identified compounds at the surface water samples (*n* = 8) and wastewater treatment plant (WWTP) effluent in Uppsala, Sweden.

The identified compounds using RP and HILIC separation techniques were divided into 6 different categories, including industrial chemicals, pharmaceuticals, personal care products, pesticides, PFASs, and natural compounds (Fig. 3, Table S6 in SI2). Industrial chemicals account for 55% of all identifications; however, this proportion mirrors the composition of our suspect list (206 of 318 entries classified as industrial; Table S1 in SI2). Consequently, raw class fractions should not be interpreted as indicators of environmental prevalence. Instead, these summaries are used to frame site-to-site differences and highlight detections uniquely enabled by HILIC^45^. Pharmaceuticals (19%) and personal care products (11%) were frequently observed within the scope of the screened suspects (Table S1 in SI2), emphasizing their resistance to wastewater removal processes^46^. The occurrence of pesticides (9.5%) (e.g., atrazine, imazapyr, diuron) indicates impacts from agricultural runoff^47^. The presence of PFAS (4.8%), an emerging group of substances, has been shown in previous studies they are ubiquitously distributed in the aquatic environment due to their high persistence and mobility^48^. In contrast, natural products remain consistently low (1.2%), suggesting natural background levels.

**Fig. 4:**
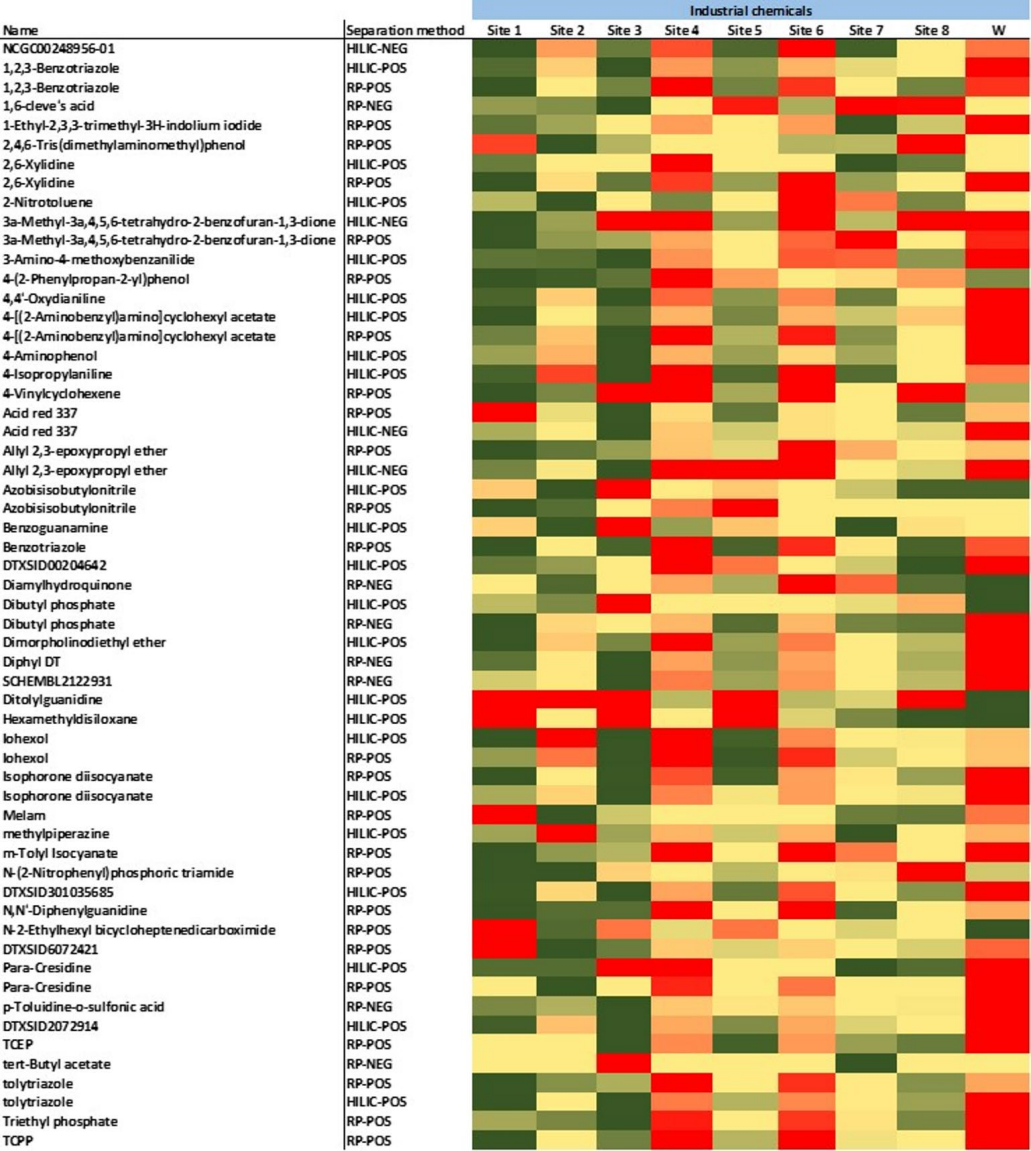
The heatmap graph of the spatial distribution of the industrial chemicals identified across diverse sampling locations.

**Fig. 5:**
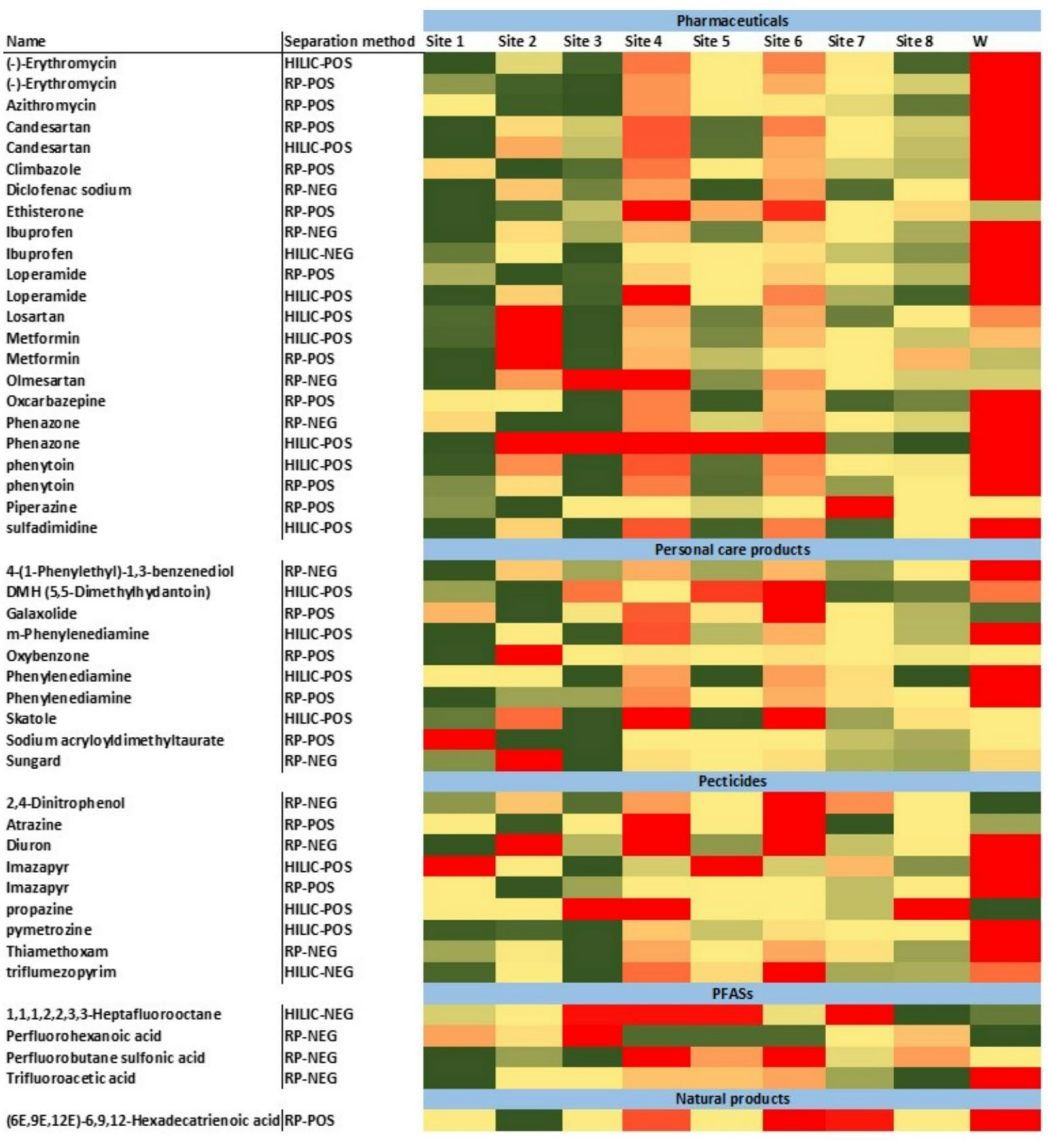
The heatmap graph of the spatial distribution of the pharmaceuticals, personal care products, pesticides, PFASs, and natural compounds identified across diverse sampling locations.

The observed presence and intensities of organic micropollutants varied largely across different sampling locations, influenced primarily by anthropogenic activities, wastewater treatment plant effluents, hydrological conditions, and physicochemical properties of the compounds^49^. As demonstrated in Figs. 4 and 5, the heatmaps of spatial distribution (cells show within-compound z-scored log₁₀ intensities: green = low, yellow = mid, red = high; gray = not detected) showed the highest intensities predominantly at locations directly impacted by WWTP effluent (site W). This site exhibited elevated contamination across all categories, specifically pharmaceuticals such as erythromycin, ibuprofen, and diclofenac, highlighting the limited removal efficiency of conventional wastewater treatment processes for pharmaceutical residues (Fig. 5)^5,50^. On the other hand, the reference site 1 was the cleanest location among all sampling sites, due to its location upstream of urban areas and upstream of WWTP effluents, which minimised exposure to contaminants commonly associated with wastewater discharge, industrial emissions, and urban runoff. Similarly, Lake Ekoln (site 7) exhibited lower contamination levels compared to the other sites, likely due to the dilution effect from diffuse sources as previously reported^5^.

In addition to the class-level heatmaps (Figs. 4 and 5), a site-to-site Spearman correlation heatmap with hierarchical clustering (Figure S6 in SI1) quantitatively supports the observed spatial patterns. Pairwise correlations coefficients ranged from approximately 0.4 to 1.0, with high within-cluster similarity (ρ ≈ 0.9–1.0) among several river and lake sites. In contrast, correlations between the WWTP effluent (W) and upstream reference sites were notably lower, for example, the W vs. Site 1 comparison showed the weakest correlation. The clustering dendrogram clearly separates WWTP-impacted locations from upstream or less-affected sites, reflecting the spatial gradient described earlier.

This structure is further supported by the NMDS ordination (Figure S7 in SI1), based on Bray–Curtis dissimilarities of Hellinger-transformed, log_10_-scaled intensities. The ordination reveals a clear separation of W from upstream references, with downstream and urban-influenced sites clustering together, and tributary sites positioned intermediately. The low stress value (reported in the figure) indicates that the two-dimensional solution effectively captures the multivariate relationships. Together, the correlation heatmap and NMDS corroborate the spatial trends inferred from the class-level results.

Locations downstream from OSSFs, such as the Husby tributary (site 3) and the Sävja River (site 4), demonstrated relatively high intensities of pharmaceuticals, including olmesartan and phenazone, as well as pesticides, such as propazine. These findings align with previous studies indicating that OSSFs often inadequately treat persistent pharmaceuticals, resulting in elevated contaminant concentrations downstream^51^.

Comparatively, upstream sites such as sites 1 and 8 generally displayed lower intensities across all categories. However, pesticide residues, notably diuron, displayed measurable intensities even at the upstream sites, suggesting agricultural runoff as a consistent diffuse source. This aligns with the understanding that pesticides primarily enter waterways through surface runoff from agricultural activities, underscoring the diffuse nature of agricultural pollution^52^.

Natural products, exemplified by hexadecatrienoic acid, maintained uniform intensities across various sites, reflecting a natural background level independent of wastewater treatment processes. In contrast, the intensities of cosmetic and personal care products were dominant at downstream urban-influenced sites 2 (e.g., oxybenzone and sunscreens) and 6 (e.g., galaxolide and skatol). This pattern correlates with human usage patterns and highlights their anthropogenic origin^53^.

Persistent substances like PFAS (e.g., perfluorohexanoic acid and perfluorobutane sulfonic acid) showed no particular distribution, indicating their ubiquitous presence in the environment due to their persistent and mobile characteristics^54^. Notably, upstream sites of the military airport Ärna Air Base (sites 1, 2, and 8) showed no significant PFAS contamination^55^.

Overall, the spatial distribution of identified organic micropollutants underscores the critical impact of WWTP discharge, industrial activities, and agricultural runoff on water quality. The significant site-specific variations align with compound properties and proximity to the source. This study demonstrates the critical role of HILIC as a valuable complementary technique to conventional RP separation methods in identifying organic micropollutants. These insights underscore the need for advanced treatment technologies and targeted pollution control measures to mitigate contaminant inputs and their environmental impacts effectively.

## Conclusion and prospects

The current study established a comprehensive LC-HESI-HRMS method for the suspect screening of PMT/vPvM substances in surface water and wastewater systems in Uppsala, Sweden. The developed HILIC separation technique, combined with RP, enabled the identification of a broad range of these chemicals, possessing diverse polarities. In total, 84 unique compounds were identified at varying confidence levels, with 27% and 48% identified solely through HILIC and RP methods, respectively. Among the identified substances, industrial chemicals (55% of identified substances, e.g., hexamethyldisiloxane and para-cresidine) and pharmaceuticals (19%, e.g., metformin and tolytriazole) emerged as the primary contaminants, exhibiting significant intensities across the investigated aquatic matrices.

This study highlights the importance of using complementary analytical methods, such as RP and HILIC, to address the limitations of single techniques, thereby ensuring a more comprehensive approach to monitoring environmental contaminants. This robust dual-separation framework can serve as a template for catchment-wide PMT/vPvM surveillance under evolving EU Water Framework Directive requirements, for stricter regulations and improved treatment technologies to manage the risks associated with PMT/vPvM substances. Additionally, ongoing environmental monitoring and effective screening methods are crucial for reducing the ecological and human health impacts of emerging aquatic pollutants.

## Supplementary Information

Below is the link to the electronic supplementary material.

Supplementary Material 2

Supplementary Material 1

## Data Availability

All data supporting the findings of this study are available within the paper and its Supplementary Information (SI), SI1 (the word file) and SI2 (spreadsheet excel file).
