# Supplementary material for: Combined RP-HILIC for suspect screening of persistent, mobile, and toxic substances in surface water: A case study
Source: Sci Rep. 2025 Dec 5;15:43272. doi: 10.1038/s41598-025-29664-1 (PMC12686432; doi:10.1038/s41598-025-29664-1)
Supplement: Supplementary file 1 — Supplementary Material 2 [file 41598_2025_29664_MOESM1_ESM.docx]

**Supplementary information (SI1)**

**Combined RP-HILIC for Suspect Screening of Persistent, Mobile, and Toxic Substances in Surface Water: A Case Study**

**Javad Mottaghipisheh*, Rajneesh Kumar Gautam, Lutz Ahrens**

*Department of Aquatic Sciences and Assessment, Swedish University of Agricultural Sciences (SLU), PO Box 7050, SE-750 07 Uppsala, Sweden*

***Corresponding author:** Javad Mottaghipisheh, E-mail: javad.Mottaghipisheh@slu.se

**Table S1.** Sampling locations with their attributes

| Location | Site | pH | Temperature | Latitude | Longitude | Date of sampling | Description |
| --- | --- | --- | --- | --- | --- | --- | --- |
| Uppsala Vatten | W | 7.122 | 19.6 | 59°50'37"N | 17°39'38"E | 21.07.2023 | Wastewater effluent, after sand filter |
| Fryis river | 1 | 7.85 | 15.5 | 60°1'59.35"N | 17°27'52.96"E | 21.07.2023 | Upstream Björklinge village before WWTP |
|  | 2 | 7.503 | 15.5 | 59°59'47.26"N | 17°31'56.34"E | 21.07.2023 | Downstream Bjorklinge-small scale WWTP |
|  | 3 | 7.974 | 16.4 | 59°52'6.20"N | 17°36'12.03"E | 21.07.2023 | Husby-OSSF site-tributary to Fyris River |
|  | 4 | 7.617 | 21.3 | 59°49'55.12"N | 17°39'38.18"E | 21.07.2023 | Sävja river-OSSF site-tributary to Fyris River |
|  | 5 | 7.668 | 19.9 | 59°49'53.63"N | 17°41'25.34"E | 21.07.2023 | Uppsala downstream large-scale WWTP |
|  | 6 | 7.609 | 22.9 | 59°48'33.76"N | 17°40'7.80"E | 21.07.2023 | After Sävja-downstream the junction of Fyris and Sävjaan river |
|  | 7 | 8.335 | 21.5 | 59°45'26.45"N | 17°38'15.86"E | 21.07.2023 | Lake Ekoln |
|  | 8 | 7.758 | 19.5 | 59°53'11.5" N | 17°34'42.5"E | 21.07.2023 | Ärnabron-upstream of the WWTP |
| Field blank |  |  |  | 59°49'55.12"N | 17°39'38.18"E | 21.07.2023 | Sampled in site 4 |
| Control Blank | | |  | | | 21.07.2023 | MQ spiked with internal standards |


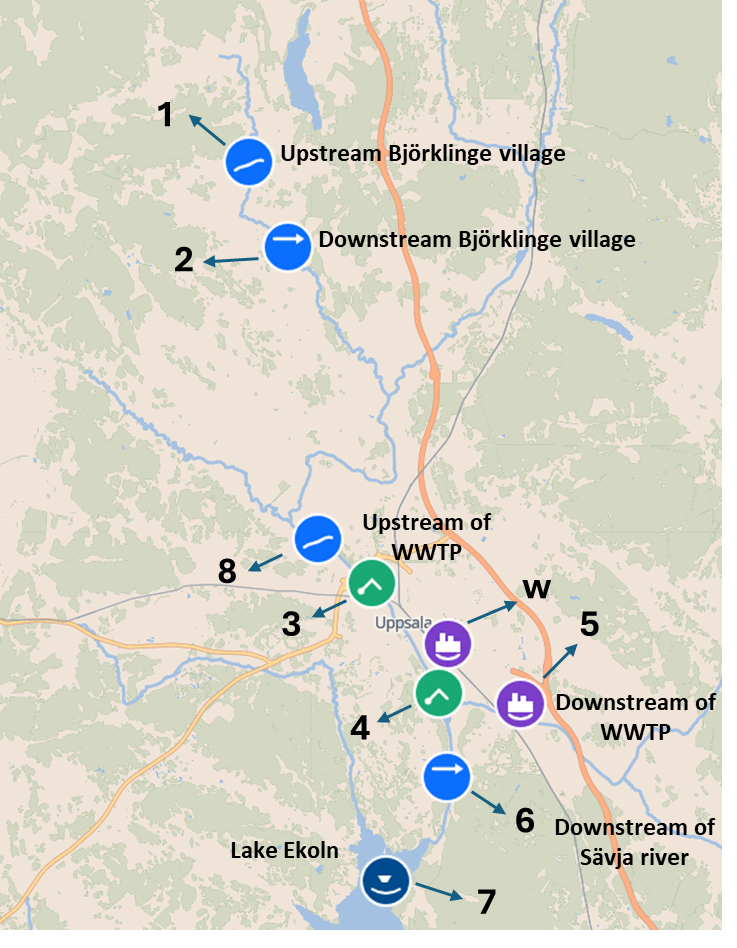
**Figure S1.** Map of Uppsala showing the locations of collected samples. Map created using OpenStreetMap data © OpenStreetMap contributors (https://www.openstreetmap.org/copyright), available under the Open Database Licence (ODbL 1.0).

**Table S2.** Internal Standards spiked into the water samples

| **Abreviated name** | **Full name** |
| --- | --- |
| M4PFBA | Perfluoro-n-[1,2,3,4-^13^C_4_] butanoic acid |
| M5PFPeA | Perfluoro-n-[1,2,3,4,5-^13^C_5_] pentanoic acid |
| M5PFHxA | Perfluoro-n-[1,2,3,4,6-^13^C_5_] hexanoic acid |
| M4PFHpA | Perfluoro-n-[1,2,3,4-^13^C_4_] heptanoic acid |
| M8PFOA | Perfluoro-n-[^13^C_8_] octanoic acid |
| M9PFNA | Perfluoro-n-[^13^C_9_] nonanoic acid |
| M6PFDA | Perfluoro-n-[1,2,3,4,5,6-^13^C_6_] decanoic acid |
| M7PFUdA | Perfluoro-n-[1,2,3,4,5,6,7-^13^C_7_] undecanoic acid |
| M2PFDoDA | Perfluoro-n-[1,2 -^13^C_2_] dodecanoic acid |
| M2PFTeDA | Perfluoro-n-[1,2-^13^C_2_] tetradecanoic acid |
| M3PFBS | Sodium perfluoro-1-[2,3,4-^13^C_3_] butane sulfonate |
| M3PFHxS | Sodium perfluoro-1-[1,2,3-^13^C_3_] hexane sulfonate |
| M8PFOS | Sodium perfluoro-[^13^C_8_] octane sulfonate |
| M8FOSA | Perfluoro-1-[^13^C_8_] octane sulfonamide |
| d3-N-MeFOSAA | N-methyl-d3-perfluoro-1-octanesulfonamidoacetic acid |
| d5-N-EtFOSAA | N-ethyl-d5-perfluoro-1-octanesulfonamidoacetic acid |
| M2-4:2 FTSA | Sodium 1H,1H,2H,2H-perfluoro-1-[1,2-^13^C_2_]-hexane sulfonic acid (4:2) |
| M2-6:2 FTSA | Sodium 1H,1H,2H,2H-perfluoro-1-[1,2-^13^C_2_]-octane sulfonate (6:2) |
| M2-8:2 FTSA | Sodium 1H,1H,2H,2H-perfluoro-1-[1,2-^13^C_2_]-decane sulfonate (8:2) |
| M3HFPO-DA | 2,3,3,3-Tetrafluoro-2-(1,1,2,2,3,3,3-heptafluoropropoxy)-^13^C_3_-propanoic acid (^13^C-GenX) |

**Table S3:** Data Processing Settings in Compound Discoverer

| **Node** | **Parameter** | **Value/Setting** |
| --- | --- | --- |
| **Input Files** | Number of Input Files |  |
|  | Spectra Selected |  |
| **Select Spectra** | Retention Time (RT) Range | 0–20 min (RP), 0–35 (HILIC) |
|  | Minimum Peak Count | 1 |
|  | Polarity Mode | Positive/Negative |
|  | S/N Threshold (FT-only) | 1.5 |
| **Align Retention Times** | Alignment Model | Adaptive curve |
|  | Maximum Shift [min] | 2 |
|  | Mass Tolerance | 5 ppm |
|  | Remove Outliers | True |
| **Detect Compounds** | Mass Tolerance | 5 ppm |
|  | Min. Peak Intensity | 1,000 |
|  | Min. # Scans per Peak | 5 |
|  | Precursor Mass Tolerance | 0.025 Da |
|  | Max. Number of Gaps to Correct | 2 |
|  | Chromatographic S/N Threshold | ≥1.5 |
|  | Remove Baseline | False |
|  | Gap Ratio Threshold | 0.35 |
|  | Max. Peak Width [min] | 1 |
|  | Isotope Pattern Detection | Br, Cl |
|  | RT Tolerance for Isotopes [min] | 0 |
|  | Use Peak Quality for Isotopes | True |
| **Group Compounds** | Mass Tolerance | 5 ppm |
|  | RT Tolerance [min] | 0.2 |
|  | Area Integration | Most Common Ion |
|  | Preferred Ions | [M+H]^+1^ / [M-H]^-1^ |
|  | Peak Rating Contributions | Area (3), CV (10), Jaggedness (5), Modality (5), Zig-Zag Index (5) |
| **Fill Gaps** | Mass Tolerance | 5 ppm |
|  | S/N Threshold | 1.5 |
|  | Use Real Peak Detection | True |
|  | Apply Restrictive Gap Filling | True |
|  | Min. # Scan per Preak | 3 |
| **Mark Background Compounds** | Max. Sample/Blank | 5 |
|  | Max. Blank/Sample | 0 |
|  | Hide Background | True |
| **Search Neutral Losses** | Neutral Losses | CO₂, HF, SO₂, CF₂, CH₂C=O, CH₃CO, etc. |
|  | High Accuracy Mass Tolerance (mmu) | 2.5 |
|  | Low Accuracy Mass Tolerance (Da) | 0.5 |
|  | S/N Threshold | 3 |
| **Assign Compound Annotations** | Mass Tolerance | 5 ppm |
|  | Data Sources | mzCloud, mzVault, ChemSpider, Predicted Compositions, Mass List |
|  | Use mzLogic, Spectral Distance | True |
| **Search mzCloud** | Precursor Mass Tolerance | 10 ppm |
|  | FT Fragment Mass Tolerance | 10 ppm |
|  | IT Fragment Mass Tolerance | 0.4 Da |
|  | Similarity Search Threshold | ≥40 |
|  | Annotate Matching Fragments | True |
| **Search mzVault** | Library | Massbank NIST |
|  | Compound Classes | All |
|  | Search Algorithm | HighChem HighRes |
|  | RT tolerance (min) | 2 |
| **Search Mass Lists** | Mass Lists | Multiple sources, including NORMAN and PFAS suspect lists |
|  | RT Tolerance [min] | 2 |
|  | Mass Tolerance | 5 ppm |
| **Search ChemSpider** | Databases | ChEMBL; ECHA; EPA DSSTox; EPA Toxcast; MassBank; PubMed |
|  | Search Mode | By formula or mass |
|  | Mass Tolerance (ppm) | 5 |
|  | Check All Predicted Composition | True |
| **Apply mzLogic** | FT Fragment Mass Tolerance (ppm) | 10 |
|  | IT Fragment Mass Tolerance (Da) | 0.4 |
|  | Max. # mzCloud Similarity Results | 10 |
|  | Match Factor Threshold | 30 |
| **Predict Compositions** | Mass Tolerance | 5 ppm |
|  | Min./Max. Element Counts | Min: C, H, O; Max: C90, H190, Br3, Cl4, N10, O18, P3, S5 |
|  | Max. RDBE | 40 |
|  | Min. Spectral Fit [%] | 30 |
|  | Min. Pattern Coverage [%] | 90 |


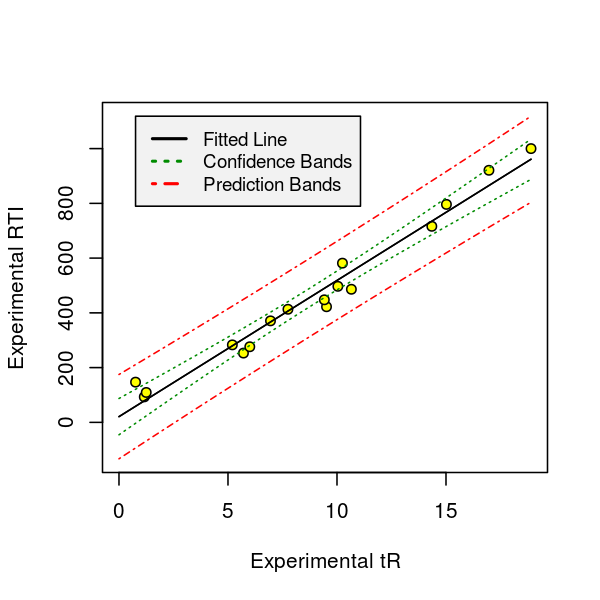


**Figure S2:** Calibration curve of calibrants in negative mode in RTI workflow


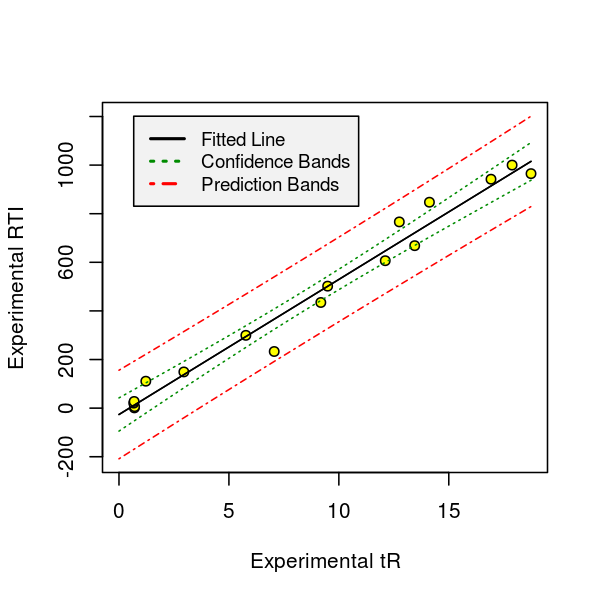


**Figure S3:** Calibration curve of calibrants in positive mode in RTI workflow

**Table S4:** Calibrant information for RTI (negative mode)

| **Compound Name** | **Molecular Formula** | **[M-H]^─^** | **[M+CH_3_COOH-H]^─^** | **[M+Cl]^─^** | **tR (min) (1st measurement)** | **tR (min) (2nd measurement)** | **tR (min) (3rd measurement)** |
| --- | --- | --- | --- | --- | --- | --- | --- |
| **Benzoic acid** | C₇H₆O₂ | 121.0295 | 181.0506 | 157.0062 | 1.16 | 1.17 | 1.16 |
| **Acephate** | C_4_H_10_NO_3_PS | 182.0046 | 242.0258 | 217.9813 | 1.24 | 1.25 | 1.25 |
| **Salicylic acid** | C_7_H_6_O_3_ | 137.0244 | 197.0455 | 173.0011 | 0.76 | 0.77 | 0.77 |
| **Simazine 2-Hydroxy** | **C₇H₁₃N₅O** | 182.1047 | 242.1259 | 218.0814 | 5.71 | 5.72 | 5.7 |
| **Tepraloxydim** | **C₁₇H₂₄ClNO₄** | 340.1321 | 400.1532 | 376.1088 | 6 | 5.92 | 5.93 |
| **Bromoxynil** | **C₇H₃Br₂NO** | 273.8509 | 333.872 | 311.8254 | 5.23 | 5.25 | 5.19 |
| **MCPA** | **C₉H₉ClO₃** | 199.0167 | 259.0368 | 234.9934 | 6.98 | 6.98 | 6.88 |
| **Valproic acid** | C₈H₁₆O₂ | 143.1078 | 203.1289 | 179.0844 | 7.77 | 7.77 | 7.71 |
| **Phenytoin** | C₁₅H₁₂N₂O₂ | 251.0826 | 311.1037 | 287.0593 | 9.55 | 9.55 | 9.51 |
| **Flamprop** | C₁₆H₁₃ClFNO₃ | 320.0495 | 380.0707 | 356.0262 | 9.45 | 9.46 | 9.38 |
| **Benodanil** | C₁₃H₁₀INO | 321.9734 | 381.9946 | 357.9501 | 10.68 | 10.68 | 10.64 |
| **Dinoterb** | C₁₀H₁₂N₂O₅ | 239.0673 | 299.0885 | 275.044 | 10.06 | 10.06 | 9.98 |
| **Inabenfide** | C₁₉H₁₅ClN₂O₂ | 337.0749 | 397.0961 | 373.0516 | 10.3 | 10.31 | 10.27 |
| **Coumaphos** | C₁₄H₁₆ClO₅PS | 361.0072 | 421.0283 | 396.9839 | 14.34 | 14.36 | 14.33 |
| **Triclosan** | C₁₂H₇Cl₃O₂ | 286.9439 | 346.965 | 324.9177 | 15.03 | 15.04 | 15.01 |
| **Avermectin B1a (Abamectin)** | C₄₈H₇₂O₁₄ | 871.4849 | 931.5061 | 907.4616 | 16.97 | 16.97 | 16.96 |
| **Salinomycin** | **C₄₂H₇₀O₁₁** | 749.4845 | 809.5057 | 785.4612 | 18.94 | 18.94 | 18.82 |

**Table S5:** Calibrant information for RTI (positive mode)

| **Compound Name** | **Molecular Formula** | **[M^+^]** | **[M+H]^+^** | **[M+H^2+^]** | **[M+NH_4_]^+^** | **[M+Na]^+^** | **tR (min) (1st measurement)** | **tR (min) (2nd measurement)** | **tR (min) (3rd measurement)** |
| --- | --- | --- | --- | --- | --- | --- | --- | --- | --- |
| **Guanylurea** | C₂H₆N₄O |  | 103.0614 |  | 120.088 | 125.0434 | 0.69 | 0.7 | 0.68 |
| **Amitrole** | C₂H₄N₄ |  | 85.0509 |  | 102.0774 | 107.0328 | 0.7 | 0.71 | 0.7 |
| **Histamine** | C₅H₉N₃ |  | 112.0869 |  | 129.1135 | 134.0689 | 0.66 | 0.67 | 0.69 |
| **Chlormequate** | C₅H₁₃ClN | 122.0731 | 123.0809 |  | 140.1075 | 145.0629 | 0.69 | 0.69 | 0.69 |
| **Methamidophos** | C₂H₈NO₂PS |  | 142.0086 |  | 159.0352 | 163.9906 | 1.21 | 1.22 | 1.21 |
| **Vancomycin** | C₆₆H₇₅Cl₂N₉O₂₄ |  | 1448.438 | 724.7224 |  |  | 2.95 | 2.95 | 2.97 |
| **Cefoperazone** | C₂₅H₂₇N₉O₈S₂ |  | 646.1497 |  | 663.1762 | 668.1316 | 7.05 | 7.06 | 7.06 |
| **Trichlorfon (Dylox)** | C₄H₈Cl₃O₄P |  | 256.9299 |  | 273.9564 | 278.9118 | 5.77 | 5.77 | 5.78 |
| **Butocarboxim** | C₇H₁₄N₂O₂S |  | 191.0849 |  | 208.1114 | 213.0668 | 14.94 | 14.94 | 14.94 |
| **Dichlorvos** | C₄H₇Cl₂O₄P |  | 220.9532 |  | 237.9797 | 242.9351 | 9.17 | 9.18 | 9.18 |
| **Tylosin** | C₄₆H₇₇NO₁₇ |  | 916.5264 |  | 933.553 | 938.5084 | 9.47 | 9.49 | 9.48 |
| **TCMTB** | C₉H₆N₂S₃ |  | 238.9766 |  | 256.0031 | 260.9585 | 12.11 | 12.11 | 12.11 |
| **Rifaximin** | C₄₃H₅₁N₃O₁₁ |  | 786.3596 |  | 803.3862 | 808.3416 | 13.46 | 13.45 | 13.46 |
| **Spinosad A (Spinosyn A)** | C₄₁H₆₅NO₁₀ |  | 732.4681 |  | 749.4947 | 754.4501 | 12.76 | 12.75 | 12.76 |
| **Emamectin B1a** | C₄₉H₇₅NO₁₃ |  | 886.5311 |  | 903.5577 | 908.5131 | 14.11 | 14.12 | 14.13 |
| **Avermectin B1a (Abamectin)** | C₄₈H₇₂O₁₄ |  | 873.4995 |  | 890.526 | 895.4814 | 16.93 | 16.92 | 16.92 |
| **Nigericin** | C₄₀H₆₈O₁₁ |  | 725.4834 |  | 742.51 | 747.4654 | 18.73 | 18.74 | 18.72 |
| **Ivermectin B1a** | C₄₈H₇₄O₁₄ |  | 875.5151 |  | 892.5417 | 897.4971 | 17.88 | 17.88 | 17.89 |

**Table S6:** Reliability categorization of the Retention Time Indices (RTI) workflow

| **Category** | **Description** |
| --- | --- |
| **Box1** | ^α^Exp. & ^β^Pred. ^γ^tR are accepted for this candidate |
| **Box2** | Although there is error, exp. & pred. tR are accepted for this candidate |
| **Box3** | Pred./Exp. tR are not reliable (high ^δ^SR; use other verification tool) |
| **Box4** | Pred./Exp. tR are not reliable (high SR; the LC method used is not well suited for this compound, it could be a false positive) |

^α^Exp.: Experimental; ^β^Pred.: Predicted; ^γ^tR: Retention time; ^δ^SR: Standardized Residuals

**Table S7:** Number of features across implementation of diverse filter steps

|  | RP-POS | RP-NEG | HILIC-POS | HILIC-NEG |
| --- | --- | --- | --- | --- |
| Total no. of features | 30986 | 19218 | 34483 | 10864 |
| After removing background | 26226 | 16674 | 30286 | 9434 |
| Peak rating ≥ 6.5 | 24833 | 16208 | 28019 | 5815 |
| Mass error: ±5 ppm | 21095 | 15009 | 26287 | 5600 |
| Area ≥ 1e5 | 20660 | 12209 | 26270 | 5599 |
| RT between 1 to 20 (RP), 1 to 30 (HILIC) | 20354 | 10519 | 19449 | 3526 |
| Exist in the mass list | 355 | 187 | 285 | 32 |
| After implementing *Criteria 1 | 58 | 35 | 40 | 7 |

***Criteria 1:** selecting the highest mzCloud, mzVault, and FISh scores, less delta mass tolerance, better peak shape, after eliminating false-positives


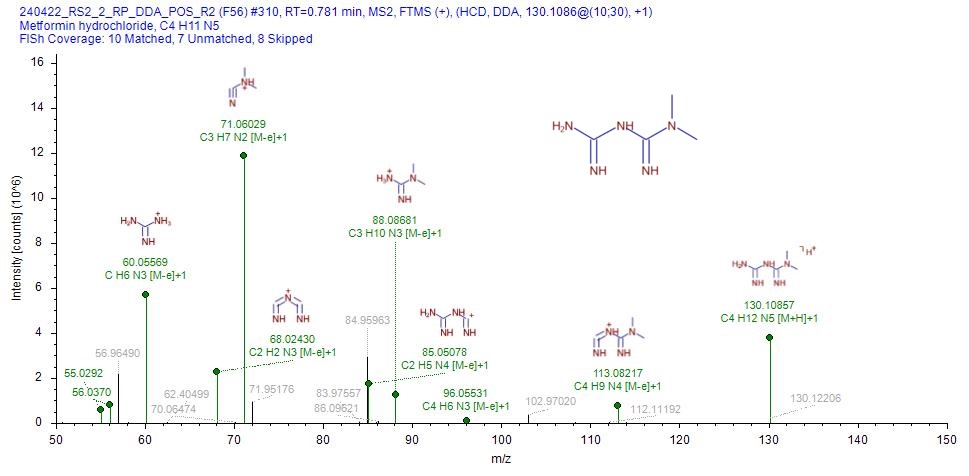


**a**

**b**


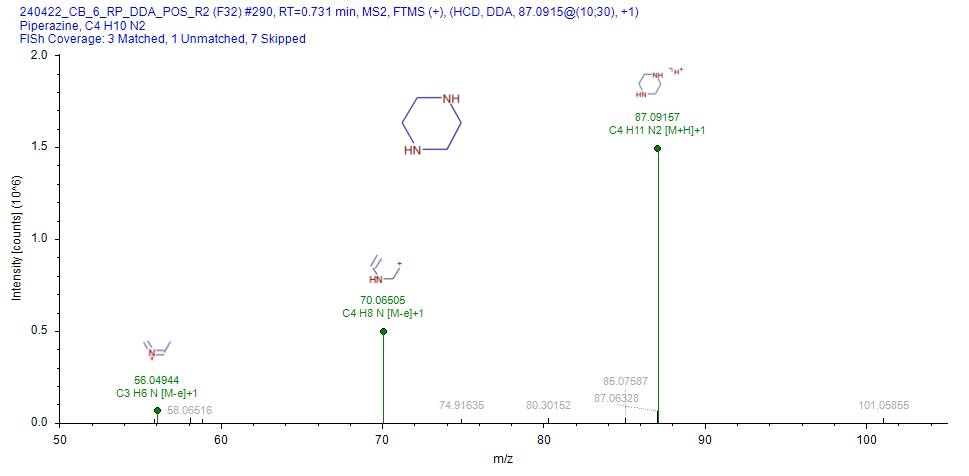


**f**

**c**

**e**

**d**

**Figure S4:** MS2 spectra of the compounds identified in level 1 a) Metformin and 2 b) Isophorone diisocyanate, c) Piperazine, d) N,N'-Diphenylguanidine, e) N-2-Ethylhexyl bicycloheptenedicarboximide, f) N-Butylbenzenesulfonamide using RP

**a**

**b**

**c**

**d**

**Figure S5:** MS2 spectra of the compounds identified in level 1 a) Metformin and 2 b) DMH (syn.5,5-Dimethylhydantoin), c) Dimorpholinodiethyl ether, d) 4-Aminophenol using HILIC

**Figure S6.** Site–site Spearman correlation heatmap of compound profiles. Cells show Spearman’s ρ between sampling sites, computed from log10(+1)–transformed intensities after collapsing HILIC and RP to one value per compound (maximum across modes). Values are printed in each cell; colors follow the scale bar. Dendrograms depict hierarchical clustering of sites using $1-\rho$as the distance. Upstream/reference locations cluster together, whereas the WWTP effluent site (W) is most dissimilar (lower ρ) to other sites.

**Figure S7.** Non-metric multidimensional scaling (NMDS) of sites using Bray–Curtis dissimilarities on Hellinger-transformed, log10-scaled intensities (RP/HILIC collapsed). Points are colored by site class and labeled; polygons show class convex hulls. WWTP effluent (W) separates from upstream references, with downstream/urban sites clustering and tributary sites intermediate (stress value shown).

**Tables in SI2 (spreadsheet excel file)**

**Table S1:** The PMT/vPvM suspect list.

**Table S2:** R script for calculating statistical analysis including Site–site Spearman correlation heatmap and Non-metric multidimensional scaling.

**Table S3:** Compounds identified using reverse phase (RP) before employing RTI.

**Table S4:** Retention Time Index (RTI)-based identification of compounds using RP after removing false-positive compounds.

**Table S5:** Compounds identified using HILIC.

**Table S6:** The combined list of compounds identified using RP and HILIC separation techniques with their correlate instensities across the samples.
